# Spotlight On Early Career Researchers: an interview with Christine Schaner Tooley

**DOI:** 10.1038/s42003-018-0208-2

**Published:** 2018-11-28

**Keywords:** Careers, Lab life, Post-translational modifications

## Abstract

Christine Schaner Tooley is an Assistant Professor at the University of Buffalo, where she studies the role of N-terminal methylation on human development and disease. In this installment of our Q&A series with early-career researchers, Christine tells us about her journey from not wanting an academic career to running her own lab, where the field is headed, and her favorite post-translational modification.

**Figure 1:**
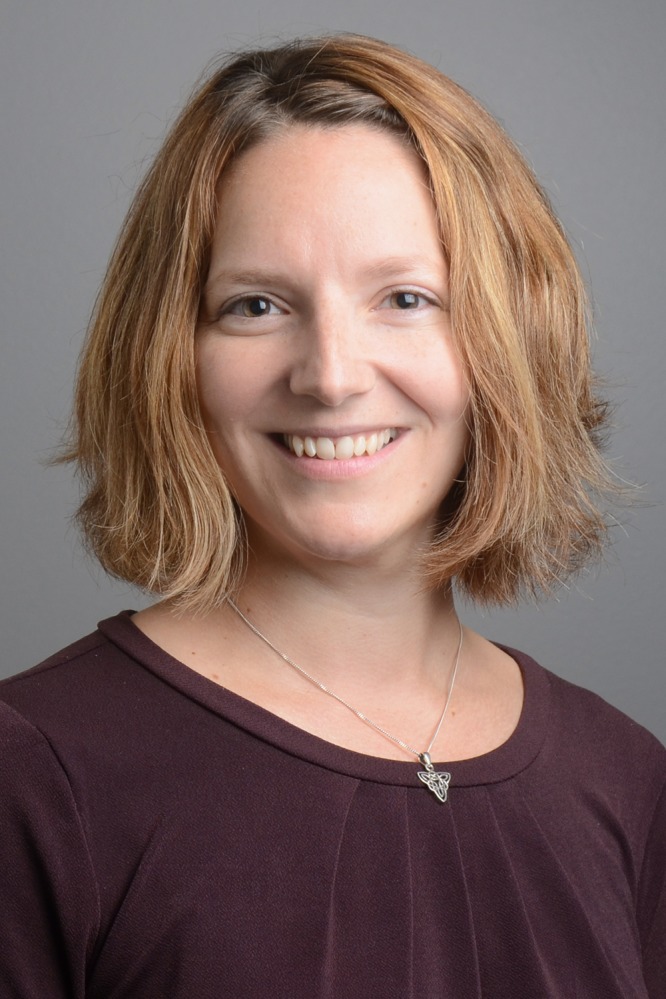
Image credit: Christine Schaner Tooley

Please tell us about your research interests**.**

My broad research interest is protein post-translational modification. I was just finishing my undergraduate education when the histone code was first hypothesized. It seemed like deciphering this code would be so interesting and so much fun. I decided to pursue histone methylation research in graduate school and have never looked back. I switched from histone methylation to N-terminal methylation when I was a postdoctoral fellow and discovered the first eukaryotic N-terminal methyltransferase. My lab now focuses on understanding the specific effects of N-terminal methylation on the function of each of its targets and the general effects of N-terminal methylation on different biological processes, such as development, cancer progression, and aging.

What has your journey been to this point?

I feel my journey has been pretty similar to many other graduate students and postdoctoral fellows that I know. I started the journey because I liked and was good at science but never had a set plan or definitive goal. I worked hard and did my best at each stage, and at the end of the each stage, I evaluated what I had produced and what opportunities this had afforded me. The only thing I thought I knew for certain was that I did not want a career in academia. I developed this thought when I was a graduate student and felt I didn’t know enough and wasn’t creative enough to lead my own research program. However, I learned that it was too early in my career to make such an assessment. By the end of my postdoc, I had developed the skills and confidence to know that I could absolutely run my own laboratory and that I very much wanted to.

What are your predictions for your field in the near future?

My immediate field of N-terminal methylation is extremely small, so I would like very much for it to expand. I hope that by proving the biological relevance of N-terminal methylation, it will lead to greater interest in determining what it does at an individual substrate level, and researchers in other fields will want to determine how N-terminal methylation affects the function of their favorite protein. In addition, I’m hoping to prove that there is interplay between different N-terminal post-translational modifications and that N-terminal modifications can affect internal post-translational modifications and function in a “code” for protein regulation. I’d like to see this type of research lead to an inclusive N-terminal post-translational modification field, so the different independent fields could work more collaboratively and gain more exposure.

Can you speak of any challenges that you have overcome?

For me, the biggest challenge has been overcoming the perception that as a woman, you can either raise a family or have a successful career in academia. This was actual advice I was given at various points of my career. I have always known I wanted to be a mother, and I think this was a big reason I initially shied away from the idea of a career in academia. It took the birth of my first son during my postdoctoral fellowship to show me that an academic career and raising children are not mutually exclusive, if you organize and work efficiently. I now have three sons and a successful career, and I hope to be an example that you can do both.

What advice would you give to your younger self?

Believe in yourself and keep moving forward. Everything works out in the end.

Which is your favorite post-translational modification and why?

Methylation, of course, it’s stable and you can always depend on it.

*This interview was conducted by Senior Editor Dominique Morneau*

